# Prevalence of Developmental Dysplasia of the Hip in Japanese Patients with Adolescent Idiopathic Scoliosis: Comparison of Conventional and Age-Adjusted Criteria

**DOI:** 10.3390/children13050709

**Published:** 2026-05-21

**Authors:** Takahiro Nishimura, Hideaki Watanabe, Naoya Taki, Ichiro Kikkawa

**Affiliations:** 1 Department of Orthopaedic Surgery, Jichi Medical University, Shimotsuke 329-0498, Japan; 2 Department of Paediatric Orthopaedics and Orthopaedic Surgery, Jichi Children’s Medical Center, Shimotsuke 329-0498, Japan; 3 Department of Orthopaedic Surgery, Nasu Central Hospital, Otawara 324-0036, Japan

**Keywords:** adolescent idiopathic scoliosis, developmental dysplasia of the hip, lateral center-edge angle, prevalence, radiographic measurement

## Abstract

**Highlights:**

**What are the main findings?**

- The prevalence of developmental dysplasia of the hip (DDH) in adolescent idiopathic scoliosis (AIS) varied substantially according to the diagnostic criteria used (5.6% with conventional criteria vs. 1.5% with age-adjusted criteria).
- Standardized standing whole-spine radiographs with exclusion of pelvic malalignment enabled more accurate assessment of acetabular morphology in patients with AIS.

**What are the implications of the main findings?**

- The estimated prevalence of DDH in AIS being strongly affected by the radiographic criteria applied highlights the importance of standardized diagnostic assessment when interpreting prevalence and considering screening strategies.
- Accurate evaluation of acetabular morphology in AIS requires careful assessment of the hip on standing whole-spine radiographs with consideration of pelvic alignment and skeletal maturity, particularly in younger adolescents.

**Abstract:**

**Background/Objectives:** The prevalence of developmental dysplasia of the hip (DDH) in adolescent idiopathic scoliosis (AIS) remains unclear, partly because of differences in diagnostic criteria and measurement accuracy. Additionally, spinopelvic alignment and skeletal maturation may affect radiographic assessment of acetabular morphology in patients with AIS. This study aimed to clarify the prevalence of DDH in Japanese patients with AIS using standardized radiographic assessment and to compare conventional and age-adjusted diagnostic criteria for DDH. **Methods:** This cross-sectional study included 602 Japanese patients aged 10–18 years with AIS. Patients with inadequate radiographs, including those with pelvic rotation and lateral inclination, were excluded to improve measurement accuracy. DDH was defined using two criteria: (1) conventional (lateral center-edge angle <20°) and (2) age-adjusted thresholds (<15° for <15 years and <18° for ≥15 years). Radiographic parameters were compared between patients with and without DDH. A multivariate logistic regression analysis was performed to identify factors independently associated with DDH. **Results:** The prevalence of DDH in AIS was 5.6% (34/602) using the conventional criterion and 1.5% (9/602) using the age-adjusted criteria. Patients with DDH showed significantly lower acetabular coverage, with a lower lateral center-edge angle and acetabular head index and higher Sharp and Tönnis angles than those without DDH (all *p* < 0.01). No significant difference in the main curve Cobb angle was observed between the groups. A younger age was independently associated with DDH, whereas sex and the main curve Cobb angle were not. **Conclusions:** The prevalence of DDH in Japanese patients with AIS varies substantially depending on the diagnostic criteria. Standardized radiographic evaluation with exclusion of pelvic rotation provides a reliable estimate. These findings highlight the importance of evaluating acetabular morphology on standing whole-spine radiographs in patients with AIS while considering skeletal maturation and spinopelvic alignment.

## 1. Introduction

Developmental dysplasia of the hip (DDH) represents a spectrum of abnormalities characterized by insufficient acetabular coverage of the femoral head, ranging from mild dysplasia to subluxation and dislocation. Radiographic assessment using the lateral center-edge angle (LCEA) is widely used, and an LCEA < 20° is commonly used to define dysplasia [1]. However, because acetabular coverage changes with growth, age-dependent thresholds have been proposed. An example of this age-dependent threshold is that an LCEA < 15° has been defined as abnormal in children younger than 15 years [2]. Additionally, an LCEA < 18° has been reported to represent clinically significant dysplasia [3]. Furthermore, DDH is recognized as a continuum of structural abnormalities, including dysplasia, subluxation, and dislocation [4]. Therefore, the definition of DDH varies across studies depending on the radiographic criteria applied.

The prevalence of DDH in the general population varies according to diagnostic thresholds and ethnicity. In Western populations, the prevalence of DDH defined by an LCEA < 20° has been reported to be approximately 4–5% [5]. In contrast, studies in Japanese populations have consistently demonstrated a higher prevalence of DDH than those in Western populations. A population-based cohort study reported a prevalence of 12.6% in Japan using an LCEA < 20° [6]. Studies using an LCEA < 25° have reported prevalence rates ranging from 5.1% to 19% in Japan, which are higher than those observed in European populations [7,8]. Furthermore, a recent Japanese community-based cohort study showed a prevalence of 21.3% using an LCEA < 25°, and up to 29.9% when multiple radiographic parameters were applied [9]. These findings indicate that DDH is common in the Japanese population, and that prevalence estimates are strongly affected by the diagnostic criteria used.

Adolescent idiopathic scoliosis (AIS) is the most common pediatric spinal deformity and is generally defined as a structural lateral curvature of the spine of at least 10° occurring near puberty [10,11]. Previous epidemiological studies have reported an AIS prevalence rate ranging from 0.47% to 5.2%, with variability according to age, sex, ethnicity, and diagnostic methodology [10]. In general, the prevalence of AIS in adolescents is estimated to be approximately 2–4%, with a female predominance [11,12]. Recent large-scale school screening studies from Japan have also suggested that the prevalence of AIS may be increasing over time. A 25-year population-based screening study from Ehime Prefecture showed that the estimated prevalence of AIS increased from 0.85% in the early study period to 2.14% in 2022 [12].

There is an association between AIS and DDH. However, the prevalence of DDH in AIS varies widely. Large database studies have reported relatively low prevalence rates of DDH in AIS, ranging from 0.24% to 0.41% [13]. In contrast, radiographic studies have reported higher prevalence rates of approximately 1.4–1.8% in general AIS populations [14]. Furthermore, studies including severe AIS have shown markedly high prevalence rates of DDH ranging from 19% to 22% [15,16].

DDH is clinically important because dysplastic morphology is associated with accelerated progression of osteoarthritis and an increased risk of total hip arthroplasty [17]. Previous long-term follow-up studies showed that hips with acetabular dysplasia frequently developed osteoarthritis before 65 years of age [18]. In Japan and other Asian countries, most cases of hip osteoarthritis are considered to be associated with acetabular dysplasia [19].

The true prevalence of DDH in AIS remains unclear because previous studies used different diagnostic criteria, including various LCEA thresholds (e.g., 18°, 20°, and 25°), and several large-scale studies were based on diagnostic coding. Additionally, many previous studies evaluated acetabular morphology using scoliosis radiographs, in which pelvic tilt and rotation may affect measurement accuracy [15,16].

Therefore, the primary aim of this study was to clarify the prevalence of DDH in Japanese patients with AIS who are younger than 18 years of age using standardized radiographic assessment. Patients with pelvic rotation were excluded to improve measurement accuracy. The secondary aim was to compare conventional and age-adjusted diagnostic criteria for DDH. This study may contribute to more appropriate screening and radiographic assessment strategies for patients with AIS.

## 2. Materials and Methods

### 2.1. Study Design and Patients

This was a single-center study conducted at a regional referral institution. This cross-sectional study was approved by the Ethics Review Board of Jichi Medical University Hospital (Bioethics Committee for Clinical Research) (Approval No.: Rinfu 20-019, approved on 20 August 2020), and was conducted in accordance with the Declaration of Helsinki (1975, as revised in 2013). The requirement for written informed consent was waived by the Ethics Review Board because of the retrospective nature of the study. Instead, an opt-out approach was used, whereby patients and their legal guardians were informed of the study by publicly available information and were provided the opportunity to decline participation.

Patients aged 10–18 years who were referred to our outpatient department for scoliosis or suspected scoliosis between February 2006 and March 2020 were eligible for inclusion. The study population was derived from a regional cohort of 371,510 individuals within a 1163-km^2^ catchment area surrounding our institution. A total of 812 patients underwent plain standing whole-spine radiography during the study period.

**Figure 1:**
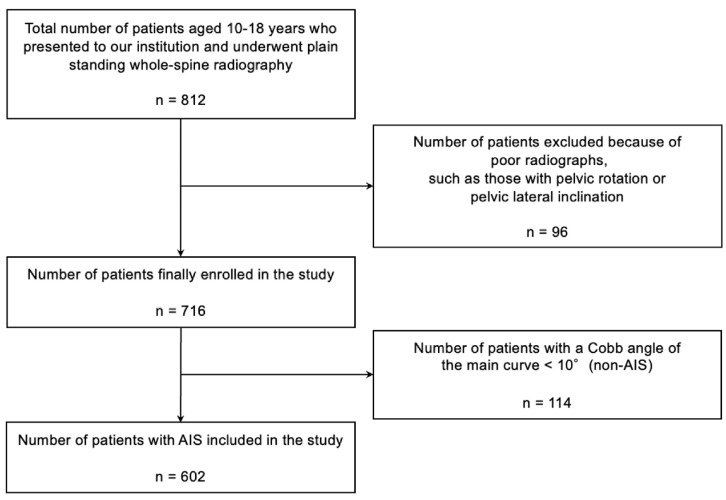
Patient selection flowchart. Flow diagram showing the selection and exclusion criteria of the patients. Patients with inadequate radiographs, including those with pelvic rotation or lateral pelvic inclination, were excluded to ensure measurement accuracy. AIS indicates adolescent idiopathic scoliosis.

After exclusion of patients with inadequate radiographic quality, including those with pelvic rotation or pelvic lateral inclination (*n* = 96), 716 patients were eligible for analysis. Among these, 602 patients met the diagnostic criteria for AIS and were included in the final analysis. Patients with a Cobb angle < 10° (*n* = 114) were excluded. None of the included patients had a history of hip pain, prior diagnosis or treatment of DDH, or inability to ambulate independently. The patient selection process is shown in Figure 1.

### 2.2. Radiographic Evaluation

All patients underwent standardized standing whole-spine radiography. To minimize measurement error, positioning of the patients was strictly controlled. The patients stood with their patellae facing forward and knees fully extended, feet shoulder-width apart, and gaze directed forward. The elbows were flexed, and both hands were placed in the supraclavicular fossae. This standardized positioning minimized pelvic rotation and improved measurement reproducibility.

**Figure 2:**
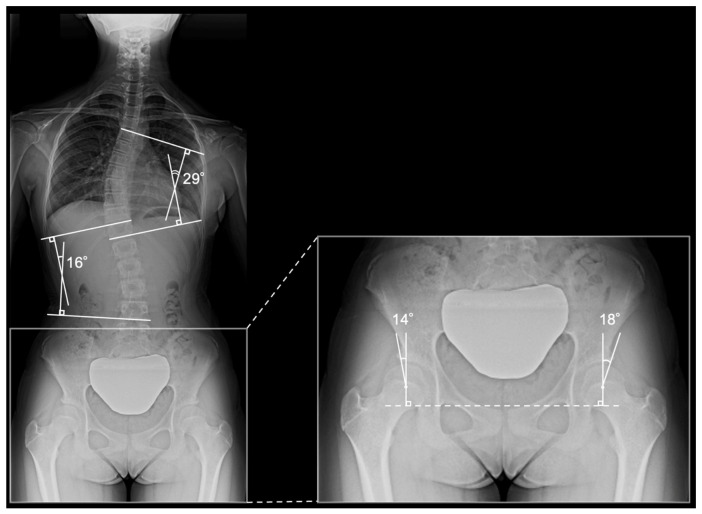
Representative radiograph and measurement of parameters. Standing whole-spine radiograph of a 12-year-old female patient without pelvic rotation. Measurements of the Cobb angle and lateral center-edge angle (LCEA) are shown. The thoracic curve measured 29°, and the thoracolumbar/lumbar curve measured 16°, which were consistent with adolescent idiopathic scoliosis. The LCEA was 14° on the right and 18° on the left. Based on a conventional cutoff of an LCEA < 20°, both hips were classified as dysplastic, whereas using age-adjusted criteria, only the right hip met the definition of developmental dysplasia of the hip.

Radiographic measurements were performed digitally by a single experienced orthopedic surgeon. The following parameters were evaluated: Cobb angle (thoracic and thoracolumbar curves), LCEA [1], Tönnis angle [20], Sharp angle [21], and acetabular head index [22]. The measurement techniques for the Cobb angle and LCEA are shown in Figure 2.

**Figure 3:**
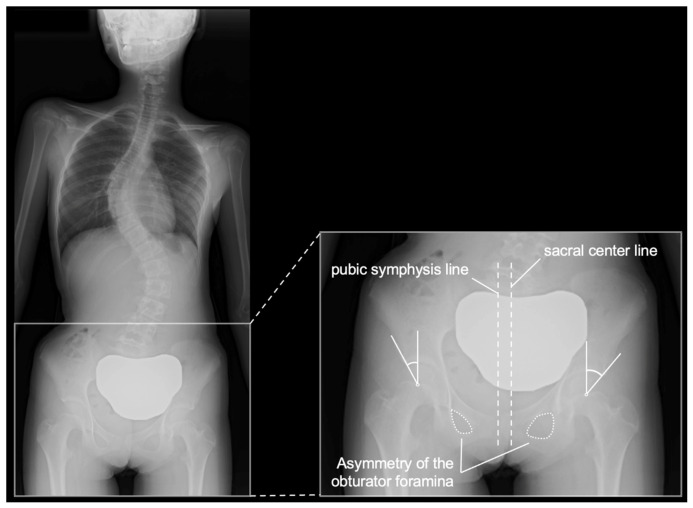
Example of an excluded radiograph owing to pelvic rotation. Standing whole-spine radiograph of a 13-year-old female patient showing pelvic rotation. Pelvic malalignment is evident based on the relative position of the sacral center and pubic symphysis, as well as asymmetry of the obturator foramina. Such rotation may result in underestimation of the lateral center-edge angle on the right side and overestimation on the left side. This case was excluded from the analysis because of compromised measurement accuracy.

Radiographs with pelvic rotation or malposition that could affect acetabular measurements were excluded from the analysis. An example of radiographs considered inappropriate for measurement because of pelvic rotation is shown in Figure 3.

### 2.3. Definitions

AIS was defined as a main curve Cobb angle ≥ 10°. DDH was defined using two different criteria: (1) a conventional criterion with an LCEA < 20°; and (2) age-adjusted criteria with an LCEA < 15° in patients younger than 15 years and an LCEA < 18° in patients aged 15 years or older. The prevalence of DDH in AIS was calculated using both definitions.

### 2.4. Statistical Analysis

Statistical analysis was performed using SPSS Version 25 (IBM Corp., Armonk, NY, USA). Radiographic parameters were compared between patients with AIS and DDH and those with AIS and normal hips using the unpaired *t*-test. A *p* value < 0.05 was considered statistically significant.

A multivariate logistic regression analysis was performed to identify factors independently associated with DDH in patients with AIS. Age, sex, and the main curve Cobb angle were included as explanatory variables. Odds ratios (ORs) and 95% confidence intervals (CIs) were calculated.

This retrospective, cross-sectional study included all eligible patients during the study period. Therefore, a formal *a priori* sample size calculation was not performed. The suitability of the multivariate model was evaluated on the basis of the events-per-variable approach. The model included 3 explanatory variables and 34 DDH events, corresponding to 11.3 events per variable.

## 3. Results

The AIS cohort consisted of 79 boys and 523 girls, with a mean age of 13.6 years. Among the 602 patients with AIS, DDH based on the conventional criterion was identified in 34 (5.6%) patients, including 13 patients with bilateral involvement. Of these, 4 were boys and 30 were girls. None of the patients had a history of hip pain, previous treatment, or prior diagnosis of DDH.

Comparisons of radiographic parameters between patients with DDH and AIS and those with normal hips and AIS are shown in Table 1. Patients with DDH and AIS showed a significantly smaller LCEA (*p* < 0.01) and acetabular head index values (*p* < 0.01) than those with normal hips and AIS, which indicated reduced acetabular coverage. In contrast, the Sharp and Tönnis angles were significantly greater in patients with DDH and AIS than in those with normal hips and AIS (both *p* < 0.01), consistent with acetabular dysplasia. There was no significant difference in the main curve Cobb angle between the two groups of patients (*p* = 0.07).

When DDH was defined using age-adjusted criteria, nine (1.5%) patients met the criteria, including two patients with bilateral involvement. Of these, one was a boy and eight were girls. Therefore, the prevalence of DDH was substantially lower when age-adjusted criteria were applied than with the application of the conventional criteria.

In the multivariate logistic regression analysis, after adjustment for sex and the main curve Cobb angle, a younger age remained significantly associated with DDH (OR, 0.75; 95% CI, 0.60–0.94; *p* = 0.014) (Table 2). In contrast, sex (OR, 1.05; 95% CI, 0.35–3.14; *p* = 0.931) was not significantly associated with DDH. Although the main curve Cobb angle showed a trend toward an association with DDH, the relationship did not reach significance (OR, 0.97; 95% CI, 0.94–1.00; *p* = 0.092).

Values are presented as the mean ± standard deviation. DDH indicates developmental dysplasia of the hip; AIS, adolescent idiopathic scoliosis; LCEA, lateral center-edge angle; AHI, acetabular head index.

## 4. Discussion

This study showed that the prevalence of DDH in Japanese patients with AIS was 5.6% when defined using a conventional LCEA threshold of <20° and 1.5% when age-adjusted criteria were applied. Using standardized radiographic assessment after exclusion of cases with pelvic lateral inclination and rotation, we found that the estimated prevalence of DDH in AIS was substantially affected by the diagnostic criteria and radiographic methodology used. These prevalence rates were intermediate between previously reported low and high estimates, suggesting that methodological heterogeneity contributes considerably to the variability among previous studies.

Previous studies have reported widely varying prevalence rates of DDH in AIS populations. Large administrative database studies have suggested relatively low prevalence rates of DDH in AIS, ranging from 0.24% to 1.4% [13,14]. However, these studies relied on diagnostic coding and may have underestimated the true prevalence by failing to capture asymptomatic or radiographically mild dysplasia. In contrast, radiographic studies have reported high prevalence rates of DDH in AIS. Fowler et al. reported a prevalence of 22% for DDH in AIS using a threshold of an LCEA < 25° [15], while Taylor et al. showed a prevalence of 19% in patients with severe AIS [16]. Notably, these studies included patients with borderline dysplasia and did not control for pelvic rotation, which may have led to overestimation of acetabular dysplasia. Additionally, studies of early-onset scoliosis have shown that hip dysplasia occurs in conjunction with a broader spectrum of musculoskeletal abnormalities, with DDH reported in 11.1% of cases and additional associated deformities frequently observed [23]. These findings suggest that scoliosis is associated with generalized musculoskeletal developmental abnormalities rather than isolated hip dysplasia.

The prevalence of DDH in AIS observed in the present study was intermediate compared with previously reported estimates. This variability among studies may not solely reflect differences in diagnostic methodology, but may also be affected by differences in spinopelvic alignment and acetabular morphology in patients with AIS. These findings suggest that factors beyond radiographic measurement techniques may contribute to variability in the reported prevalence of DDH in AIS.

The relationship between AIS and DDH may be explained not only by radiographic alignment changes but also by developmental and biomechanical interactions involving the spine, pelvis, and hip. Previous studies have suggested that musculoskeletal deformities may originate early in development, even prenatally. De Maio et al. showed pathogenetic mechanisms underlying congenital skeletal deformities based on prenatal pathological findings in tibial bowing [24]. Furthermore, previous studies have reported a high prevalence of associated musculoskeletal conditions, including hip dysplasia, torticollis, plagiocephaly, metatarsus adductus, and clubfoot, in patients with idiopathic early-onset scoliosis [23]. These findings suggested generalized developmental or postural abnormalities rather than isolated spinal deformity, and that AIS and DDH share certain developmental predispositions involving the musculoskeletal system. Therefore, AIS and DDH may represent partially overlapping manifestations of broader developmental and biomechanical abnormalities, although the precise underlying mechanisms remain unclear.

In addition to developmental predisposition, acetabular morphology changes dynamically during growth and skeletal maturation. Than et al. showed that radiographic hip morphology continues to develop throughout childhood and adolescence, with the lateral center-edge angle increasing progressively after 10 years of age and the rate of change slowing after approximately 15 years [25]. Their findings indicate that acetabular coverage is not a fixed anatomical parameter during adolescence. This developmental process may be particularly relevant in AIS, which commonly progresses during the adolescent growth spurt. Therefore, the use of fixed adult radiographic thresholds may overestimate dysplasia in younger adolescents.

AIS has also been increasingly recognized as a whole-body postural and biomechanical disorder rather than an isolated spinal deformity. Previous studies have shown that patients with AIS have altered trunk balance, asymmetrical loading, compensatory pelvic and lower-extremity mechanics, and gait asymmetry [26,27]. Three-dimensional analyses further showed that patients with AIS showed asymmetrical acetabular morphology, with relative undercoverage on the elevated side and overcoverage on the lowered side [28]. Additionally, both hips showed increased acetabular anteversion and decreased anterior coverage. These changes are strongly associated with pelvic tilt, indicating that pelvic orientation plays a central role in acetabular coverage. Furthermore, asymmetrical pelvic obliquity in AIS leads to side-specific differences in acetabular coverage, with relative undercoverage on the elevated side. Sklensky et al. showed that approximately two thirds of patients with AIS showed asymmetric hip loading during gait, suggesting persistent asymmetric biomechanical loading during growth [29]. Increased hip loading is associated with cartilage degeneration and the development of osteoarthritis, indicating that biomechanical alterations in AIS may have long-term clinical implications.

In addition to these structural and functional changes, DDH alone may further affect pelvic alignment. Zhao et al. reported that patients with AIS and concomitant hip dysplasia showed increased pelvic obliquity, sacral tilt, and sacroiliac discrepancy, suggesting that hip dysplasia exacerbates coronal imbalance [30]. Furthermore, in their study, a strong correlation was observed between iliac obliquity and sacroiliac discrepancy, suggesting that hip dysplasia contributes to pelvic imbalance in AIS. These findings support a bidirectional relationship in which spinal deformity affects acetabular morphology, while hip dysplasia further alters pelvic alignment. This concept is also supported by studies on hip-spine syndrome, showing a high frequency of spinal anomalies in patients with acetabular dysplasia and emphasizing the importance of evaluating the lumbar–pelvic–femoral complex as an integrated biomechanical unit [31].

Nielsen et al. reported that acetabular parameters changed following posterior spinal fusion in patients with spinal deformity, suggesting that acetabular orientation is not static but is affected by spinopelvic alignment [32]. Ippolito et al. showed that skeletal deformities may evolve dynamically during growth under biological and biomechanical influences, indicating that bone morphology is continuously remodeled over time rather than remaining static [33]. The variability in the prevalence of DDH observed in AIS may not solely reflect diagnostic inconsistency, but also the dynamic interaction between growth-related skeletal remodeling and altered biomechanical loading. These findings suggest that acetabular morphology and hip coverage in AIS change over time during skeletal maturation.

Radiographic assessment of acetabular morphology is primarily affected by pelvic orientation, particularly pelvic rotation and tilt. Even small changes in pelvic alignment can produce clinically meaningful differences in the LCEA and other acetabular parameters [34,35]. Measurement error increases nonlinearly with increasing pelvic malposition, especially for parameters related to acetabular version [36]. In contrast, radiographic acquisition parameters, including beam center position and source-to-detector distance, appear to have a relatively limited effect on acetabular measurements [36,37]. Collectively, these findings suggest that pelvic orientation is the major determinant of measurement accuracy.

In this study, patients with pelvic rotation were excluded to minimize positional distortion and improve the reliability of acetabular measurements. Although standing whole-spine radiographs were used instead of dedicated pelvic radiographs, the longer source-to-detector distance and wider field of view in our institutional protocol may have reduced angular distortion and preserved reasonable measurement reliability.

Recent three-dimensional analyses further showed that the LCEA remained relatively stable within the physiological range of pelvic tilt, but decreased progressively with excessive pelvic retroversion or anteversion [38]. These findings indicate that abnormal pelvic alignment associated with AIS may substantially affect acetabular morphology and assessment of DDH.

These considerations are clinically important because variability in the reported prevalence of DDH in AIS may reflect not only methodological differences but also altered spinopelvic alignment and growth-related biomechanical remodeling during adolescence. Therefore, accurate recognition of DDH in AIS may be relevant for identifying patients at risk of hip dysfunction or secondary osteoarthritis, and may support careful hip assessment and follow-up in selected patients with AIS.

This study has several limitations. First, hip parameters were measured using standing whole-spine radiographs rather than dedicated pelvic radiographs. Although this approach reflects real-world clinical practice, differences in imaging protocols may have affected measurement accuracy. Second, patients with pelvic inclination or rotation were excluded to improve measurement precision and minimize positional distortion of acetabular morphology. However, this exclusion criterion may have resulted in selection bias by excluding patients with more severe deformity or spinopelvic imbalance, potentially leading to underestimation of the true prevalence of DDH in AIS. Additionally, the study population consisted predominantly of female patients, reflecting the epidemiology of AIS, but potentially limiting generalizability.

Third, spinopelvic alignment parameters, particularly sagittal parameters, were not evaluated in the present study. Lateral radiographs were not available for all patients, and sagittal alignment measurements were not routinely obtained. Therefore, a detailed analysis of sagittal spinopelvic parameters could not be performed. Future studies incorporating comprehensive sagittal alignment evaluation are required to further clarify the relationship between spinopelvic balance, skeletal growth, and acetabular morphology in AIS.

Fourth, all measurements were primarily performed by a single observer, which may have introduced observer-related bias. However, our previous study using the same standing whole-spine radiographic protocol showed good reliability for LCEA measurements, with an intra-rater intraclass correlation coefficient of 0.87 and an inter-rater intraclass correlation coefficient of 0.80 [39]. These findings suggest acceptable reproducibility of the measurement method, although observer-related bias cannot be completely excluded.

Fifth, although a multivariate logistic regression analysis was performed, the number of patients with DDH remained relatively limited. Therefore, additional subgroup analyses according to the severity of scoliosis or detailed spinopelvic morphology would have lacked sufficient statistical power and thus were not performed in the present study. Future multicenter studies with larger cohorts are required to allow more comprehensive stratified analyses and validation of the present findings.

Despite these limitations, this study provides a methodologically standardized estimation of the prevalence of DDH in AIS by addressing several important limitations of previous studies. Our findings suggest that the coexistence of AIS and DDH may be affected not only by radiographic assessment methods but also by developmental, growth-related, structural, and biomechanical factors involving the spine–pelvis–hip complex.

## 5. Conclusions

This study showed that the prevalence of DDH in Japanese patients with AIS was 5.6% using the conventional criterion and 1.5% using age-adjusted criteria. These findings suggest that the estimated prevalence of DDH in AIS is highly dependent on diagnostic thresholds. Using standardized radiographic evaluation and excluding cases with pelvic rotation, this study provides a more accurate assessment of acetabular morphology in AIS than previous studies. Our findings highlight the importance of evaluating acetabular morphology on standing whole-spine radiographs in patients with AIS while considering skeletal maturation and spinopelvic alignment.

## Figures and Tables

**Table 1:** Comparison of radiographic parameters between patients with DDH and AIS and those with normal hips and AIS.

	DDH and AIS (*n* = 34)	Normal Hips and AIS (*n* = 568)	*p*
LCEA (°)	17.6 ± 1.7	28.8 ± 4.7	<0.01
Sharp angle (°)	48.6 ± 3.0	44.0 ± 3.1	<0.01
Tönnis angle (°)	11.4 ± 2.5	4.9 ± 3.5	<0.01
AHI (%)	72.0 ± 5.2	82.7 ± 5.1	<0.01
Main curve Cobb angle (°)	23.6 ± 9.1	27.3 ± 12.0	0.07

**Table 2:** Multivariate logistic regression analysis of factors independently associated with DDH in patients with AIS.

Variable	Odds Ratio	95% CI	*p*
Age	0.75	0.60–0.94	0.014
Sex (girl)	1.05	0.35–3.14	0.931
Main curve Cobb angle	0.97	0.94–1.00	0.092

DDH indicates developmental dysplasia of the hip; AIS, adolescent idiopathic scoliosis; CI, confidence interval.

## Data Availability

The datasets used and/or analyzed during the current study are available from the corresponding author on reasonable request. The data are not publicly available due to privacy and ethical restrictions related to patient information; however, anonymized data may be available from the corresponding author upon reasonable request and with institutional approval.
